# YTHDF1 Aggravates the Progression of Cervical Cancer Through m^6^A-Mediated Up-Regulation of RANBP2

**DOI:** 10.3389/fonc.2021.650383

**Published:** 2021-03-19

**Authors:** Haocheng Wang, Qingya Luo, Jianyi Kang, Qinglv Wei, Yu Yang, Dan Yang, Xiaoyi Liu, Tao Liu, Ping Yi

**Affiliations:** ^1^ Department of Obstetrics and Gynecology, The Third Affiliated Hospital of Chongqing Medical University, Chongqing, China; ^2^ Department of Pathology, The First Affiliated Hospital of Army Medical University, Chongqing, China; ^3^ Research Institute of Surgery, Daping Hospital, Army Medical University, Chongqing, China

**Keywords:** cervical cancer, N6-methyladenosine, YTHDF1, tumorigenicity, RANBP2

## Abstract

N6-methyladenosine (m^6^A) is the most common post-transcriptional modification of RNA in eukaryotes, which has been demonstrated to play important roles in various cancers. YTHDF1 acts as a crucial m^6^A “reader” and regulates the fate of m^6^A modified mRNA. However, its role in cervical cancer remains unknown. In this study, we showed that YTHDF1 was highly expressed in cervical cancer, and was closely associated with the poor prognosis of cervical cancer patients. YTHDF1 knockdown suppressed the growth, migration and invasion, and induced apoptosis of cervical cancer cells. Moreover, YTHDF1 knockdown inhibited tumorigenesis of cervical cancer cells *in vivo*. Through combined on-line data analysis of RIP-seq, meRIP-seq and Ribo-seq upon YTHDF1 knockdown, RANBP2 was identified as the key target of YTHDF1 in cervical cancer cells. YTHDF1 regulated RANBP2 translation in an m^6^A-dependent manner without effect on its mRNA expression. RANBP2 potentiated the growth, migration and invasion of cervical cancer cells. Our study demonstrated the oncogenic role of YTHDF1 in cervical cancer by regulating RANBP2 expression and YTHDF1 represents a potential target for cervical cancer therapy.

## Introduction

Cervical cancer (CC) is one of the most common malignant tumors among female patients, which mortality rate ranks fourth in the world (1). Contrary to developed countries, the incidence and death rate of cervical cancer in China are increasing year by year, which seriously threatens women’s health and life (2). Although HPV vaccine is conducive to reducing the morbidity and mortality of cervical cancer, the prognosis of patients with advanced cervical cancer remains poor (3). Therefore, finding new potential targets is required for cervical cancer treatment.

N6-methyladenosine (m^6^A) is the most common post-transcriptional modification of RNA in eukaryotes (4). By regulating gene splicing (5), RNA stability (6), RNA editing (7) and mRNA translation (8), it extends the role of m^6^A regulation to almost all important biological processes (9), including animal development (10) and various human diseases (11). Increasing studies have shown that the m^6^A regulators including m^6^A “writers”, “erasers” and “readers” are dysregulated in multiple cancers and play an important role in tumor cell proliferation, differentiation arrest, survival, tumorigenesis and metastasis (12). For example, METTL3 is highly expressed in acute myeloid leukemia (AML) cells. METTL3 not only activates the oncogene c-MYC by enhancing the m^6^A modification of SP1 (13), it also promotes the translation of BCL2 and PTEN mRNA through the up-regulation of their m^6^A modification (14), which ultimately leads to the development of AML. The increase of YTHDF2 expression also promotes the proliferation of leukemia cells (15). YTHDC2 enhances mRNA translation and up-regulates the expression of metastasis-related proteins such as HIF1A, thereby inducing colon cancer metastasis (16). In breast cancer, FTO promotes cancer cell proliferation and metastasis through negative regulation of BNIP3 mRNA, and the high level of FTO is associated with the poor prognosis (17). Therefore, research on m^6^A will help to find new targets for the treatment of malignant tumors. YTHDF1 acts as a crucial m^6^A “reader” and regulates m^6^A modified mRNA translation (18, 19). Recently, YTHDF1 has been reported to be associated with the occurrence and development of several cancers. YTHDF1 promotes the epithelial-mesenchymal transition (EMT) of liver cancer cells by regulating the translation of Snail mRNA (20). In ovarian cancer, YTHDF1 promotes cancer progression by enhancing EIF3C translation (21). YTHDF1 is up-regulated in human colon cancer tissues, which predicts the poor prognosis of colon cancer patients (22).

In this study, we show that YTHDF1 is highly expressed in cervical cancer, and is closely associated with the poor prognosis of cervical cancer patients. YTHDF1 is indispensable for the proliferation and metastasis of cervical cancer cells. Combined with on-line data analysis, RANBP2 is identified as the key target of YTHDF1 in cervical cancer cells. Together, our study demonstrates the oncogenic role of YTHDF1 in cervical cancer by regulating the expression of RANBP2.

## Materials and Methods

### Tumor Samples

Cervical squamous cell carcinoma and normal cervical tissue specimens were from patients undergoing surgery in Daping Hospital of the Army Military Medical University from 2020-03-01 to 2020-05-30. All these specimens were pathologically verified, all subjects were informed consent, and the institutional review board of Daping Hospital of the Army Military Medical University approved the study (AMUWEC20190196).

### Gene Expression and Survival Analysis in Cervical Cancer Datasets

Expression data were downloaded from the Oncomine (https://www.oncomine.org/), cBioPortal (http://www.cbioportal.org/), and GEO datasets (www.ncbi.nlm.nih.gov/geo). Data was processed by GraphPad Prism 7 and processed data is in supplementary material.

### Cell Culture

HEK293T, Hela and Siha cells were purchased from National Infrastructure of Cell Line Resource (Beijing, China) and long-term stored in liquid nitrogen. HEK293T and Hela cells were cultured in DMEM (GIBCO, USA) and Siha cells were cultured in MEM (GIBCO, USA) medium. The medium was supplemented with 10% fetal bovine serum (FBS; GIBCO, USA), penicillin (100 U/ml; GIBCO, USA) and streptomycin (200 g/ml; GIBCO, USA). All the cells were maintained at 37°C in 5% CO_2_ cell culture incubator.

### Plasmids

Synthesized the complementary nucleotide sequence of shRNAs targeting YTHDF1 or RANBP2, and clone into PLKO.1 vector (#10878, Addgene) after respectively annealing. The related sequences of shRNAs were shown in **Supplementary Table**. YTHDF1-wt (YTHDF1-FLAG) and YTHDF1-mut (K395A, Y397A) expression plasmids were cloned into pCMV6 vector (OriGene, USA), and were transfected into cervical cancer cells with a lentivirus-mediated method.

### Lentiviral Infection

Plasmids and lentiviral vectors were transfected into HEK293T cells with packaging vectors psPAX2 (#12260, Addgene) and pMD2.G (#12259, Addgene) using lipofectamine LTX (Invitrogen, USA). Infectious lentivirus particles were harvested at 48 hours after transfection.

### RNA Isolation and RT-qPCR

Total RNA was extracted from cervical cancer cells using Trizol (Sigma, USA) according to the manufacturer’s instruction. RNA was reversely transcribed to cDNA by using All-in-One cDNA Synthesis SuperMix (Bimake). qPCR assays were performed in QuantStudioDx instrument (Life Technologies) following the manufacturer manufacturer following ChamQ Universal SYBR qPCR Master Mix (Vazyme). All samples were normalized to GAPDH. Primers used in RT-qPCR were listed in **Supplementary Table**.

### Western Blot

Hela and Siha cells were collected and lysed with cell lysis buffer for western blot and IP (Beyotime, China) supplemented with protease inhibitor cocktail (APExBIO, USA) to harvest proteins. Cells were lysed on ice for 30 min, and the lysate was obtained by centrifugation at 12,000 rpm for 15 min. Proteins were fractionated by SDS-PAGE, and then transferred onto 0.45 μM PVDF membranes. The PVDF membranes were blocked with 5% nonfat milk in TBS/Tween-20, and blotted with specific antibodies at 4°C overnight. The antibodies used for western blot are as follows: YTHDF1 (ProteinTech, 1:1000), RANBP2 (ProteinTech, 1:1000), GAPDH (ZSGB-BIO, 1:2000), Flag-tag (MBL, 1:5000). After washed with TBS/Tween-20, the PVDF membranes were incubated with fluorescent secondary antibody (LI-COR, IRDye 680RD Goat anti-Rabbit and IRDye 800CW Goat anti-Mouse, 1:10000). ODYSSEY Clx Two-color infrared laser imaging system (LI-COR, USA) was used to visualize the bands.

### Immunohistochemistry

Conventional paraffin sections were deparaffinized and dehydrated. After antigen retrieved, citric acid buffer (ZSGB-BIO, ZLI-9064, pH 6.0) was used to inactivate endogenous peroxidase (ZSGB-BIO, PV-9001). The slices were washed three times with PBS, and blocked with goat serum (ZSGB-BIO, ZLI-9021) at 37°C for 30 min. Rabbit anti-YTHDF1 antibody (ProteinTech, 1:100) or anti-RANBP2 antibody (ProteinTech, 1:100) was added to the slices and incubated overnight at 4°C. Next day, Goat anti-rabbit IgG (ZSGB-BIO, PV-9001) was added to the slices and incubated at 37°C for 40 min. DAB reagent (ZSGB-BIO, ZLI-9018) was used to develop color. The sections were stained with hematoxylin, decolorized with hydrochloric acid and ethanol, dehydrated and then mounted. The comprehensive score was calculated independently by two professional pathologists.

### Cell Growth and Proliferation Assays

The cells were seeded in 96-well (1000 Hela cells or 2000 Siha cells each well) plates for the cell viability test. CCK-8 reagent (DOJINDO, Japan) was added into the plate and incubated at 37°C for 2 hours. The cell absorbance at 450 nm and 630 nm wavelengths was measured by using the microplate reader (BioTek, USA) at 0 hour, 24 hours, 48 hours, 72 hours and 96 hours. All experiments were performed in triplicate.

For the colony formation assay, Hela cells were plated with 1,000 cells per well, and Siha cells were plated with 2,000 cells per well, and the medium was changed every 4 days. Hela and Siha cells were cultured for 7 days and 10 days, respectively, fixed with 4% paraformaldehyde, then stained with 0.1% crystal violet (Sigma-Aldrich, USA) for 1 hour. The number of colonies containing more than 50 cells was counted.

### Apoptosis Assay

5 × 10^5^ Hela or Siha cells were starved for 24 hours, trypsinized (without EDTA), and then stained with Annexin V/PI Apoptosis Kit (DOJINDO, Japan). The stained cells were subjected to flow cytometry. BD Accuri C6 flow cytometer (BD Biosciences, USA) was used to analyze apoptosis and all gates were drawn based on fluorescence minus one (FMO).

### Transwell Migration and Invasion Assays

Millicell Hanging Cell Culture 24 well PET 8 μm 48/pk (Millipore, Germany) was used for migration and invasion assays. For migration analysis, 6 × 10^4^ Hela or 8× 10^4^ Siha cells were diluted with 0.2 ml serum-free medium and inoculated in the upper room. 0.6 ml culture media with 20% FBS was added in the lower chamber, then incubated at 37°C for 24 hours. Transwell chambers were fixed with 4% paraformaldehyde for 30 min, and then stained with 0.1% crystal violet (Sigma-Aldrich, USA). For invasion assays, 50 μl of serum-free medium containing 10% Matrigel (Sigma-Aldrich, USA) was added in the upper chamber in advance, and then inoculated cells after it solidified.

### Animal

The animal studies were approved by the Institutional Animal Care and Use Committee of Daping Hospital affiliated to Army military Medical University, and carried out according to institutional guidelines. Hela cells infected with shRNAs or empty vector were collected and resuspended in DMEM without FBS. Balb/c female nude mice at 4-6 weeks old were injected with 4 × 10^6^ cells subcutaneously on the back. The female nude mice were sacrificed at 23 days and the tumor weight was measured.

### RNA Immunoprecipitation (RIP)

2 × 10^7^ Hela or 3 × 10^7^ Siha cells were collected in a prepared IP lysis buffer (HEPES 20 mM, 150 mM NaCl, 10 mM KCl, 5 mM EDTA, 5 mM MgCl_2_, 0.5% NP40, 10% glycerol). After incubation for 30 min at 4°C, the lysate was harvested by centrifugation at 12 000 rpm for 10 min. The supernatant lysate was incubated overnight at 4°C with 3 μg antibody. Dynabeads Protein A beads (Invitrogen, USA) were added into the lysate and incubated at 4°C for 4 hours. After washed for three times, the co-precipitated RNA was extracted with Trizol (Sigma, USA) reagent. RNA isolation and RT-qPCR were performed as described previously.

### Methylated RNA Immunoprecipitation (meRIP) and RT-qPCR

3 × 10^7^ Hela or 4 × 10^7^ Siha cells were prepared for RNA extraction. Total RNAs were extracted by using Trizol (Sigma, USA). mRNA was extracted by using PolyATtract^®^ Systems IV (Promega, USA) kit. The co-precipitated RNA was obtained according to the standard protocol of the Magna MeRIP m^6^A Kit (Merck Millipore, Germany). Briefly, 50 μl protein A/G beads were incubated with 2.5 μg anti-m^6^A antibody for 30 min at room temperature. Then 5 μg fragmented mRNA was incubated with anti-m^6^A antibody coated protein A/G beads at 4°C overnight immunoprecitation. After washes, RNA isolation and RT-qPCR were performed as described previously.

### Statistical Analysis

Statistical computations were performed using GraphPad Prism 7. A t-test was performed to compare the differences between the two groups. The growth rates difference was determined by ANOVA with repeated measures analysis of variances. The correlation between YTHDF1 expression and DNA methylation, and the correlation between YTHDF1 and RANBP2 expression in cervical cancer were analyzed by Spearman rank correlation analysis. The results were considered statistically significant when P < 0.05.

## Results

### YTHDF1 Is Highly Expressed in Cervical Cancer

**Figure 1:**
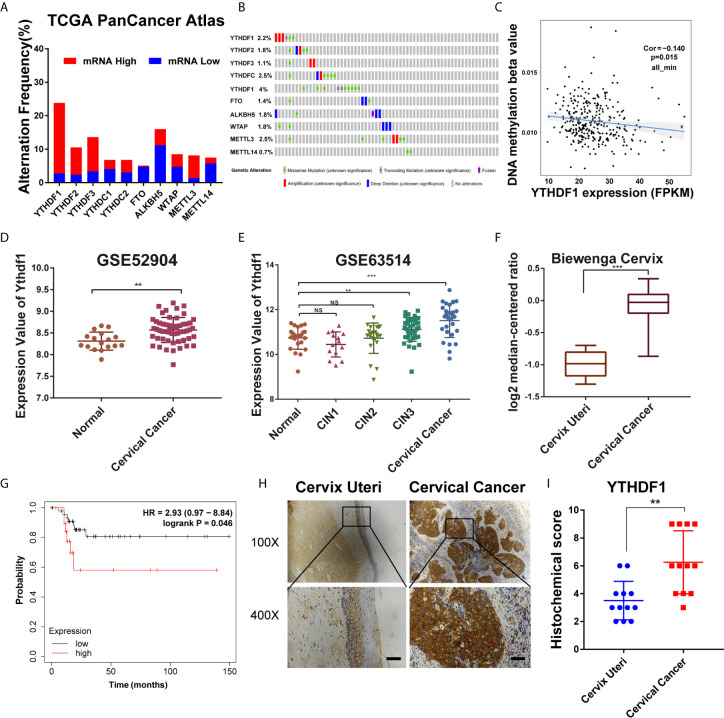
YTHDF1 is highly expressed in cervical cancer. **(A, B)** Gene expression and gene mutation rates of m^6^A-associated genes in cervical cancer according to cBioPortal dataset (TCGA PanCancer Atlas). **(C)** Correlation analysis between gene expression and DNA methylation beta value. **(D, E)** Relative RNA levels of YTHDF1 in cervical cancer and normal cervical epithelium in GEO datasets (GSE52904 and GSE63541). **(F)** Relative RNA levels of YTHDF1 in cervical cancer and cervix uteri in Oncomine datasets (Biewenga Cervix). **(G)** Kaplan-Meier analysis of RFS based on YTHDF1 expression according to KM plotter (n=68). **(H)** Representative immunohistochemical images of YTHDF1 expression in cervical cancer tissues and cervical epithelium tissues. Scale bar, 100 μm. **(I)** The quantitative analysis of YTHDF1 expression in cervical cancer tissues and cervical epithelium tissues assessed by immunohistochemistry. Data are shown as means ± S.D. **P < 0.01, ***P < 0.001, NS, not significant.

To examine the role of m^6^A regulators in cervical cancer, cBioPortal database (http://www.cbioportal.org/) was used to analyze the expression level of m^6^A regulators in cervical cancer. Results showed that the expression of several m^6^A regulators was changed in cervical cancer, among which YTHDF1 expression was increased most significantly (**Figure 1A**). Intriguingly, the DNA copy number of YTHDF1 did not change substantially (**Figure 1B**). We investigated the DNA methylation at the YTHDF1 promoter and found that DNA methylation was negatively correlated with YTHDF1 expression in cervical cancer (**Figure 1C**). Thus the high expression of YTHDF1 in cervical cancer may be partially due to DNA hypomethylation. By analyzing the two GEO datasets (GSE63514 and GSE52904) (**Figures 1D, E** **)** and the Biewenga Cervix dataset of the Oncomine database (**Figure 1F**), we found that YTHDF1 was highly expressed in cervical cancer compared to normal cervical epithelial cells. Moreover, we performed Kaplan-Meier survival analysis and found that cervical cancer patients with high expression of YTHDF1 had the poor recurrence-free survival (RFS) (**Figure 1G**). In order to further confirm the expression of YTHDF1 in cervical cancer, immunohistochemistry (IHC) assay was used to detect the expression of YTHDF1 protein in 10 pairs of cervical cancer and normal cervical epithelial tissues. Results showed that the expression level of YTHDF1 in cervical cancer was higher than that in normal cervical epithelium (**Figures 1H, I** **)**. Taken together, these data demonstrated that the m^6^A reader YTHDF1 is highly expressed in cervical cancer and is related to the poor prognosis of cervical cancer patients.

### YTHDF1 Regulates the Proliferation, Apoptosis, Migration, and Invasion of Cervical Cancer Cells

**Figure 2:**
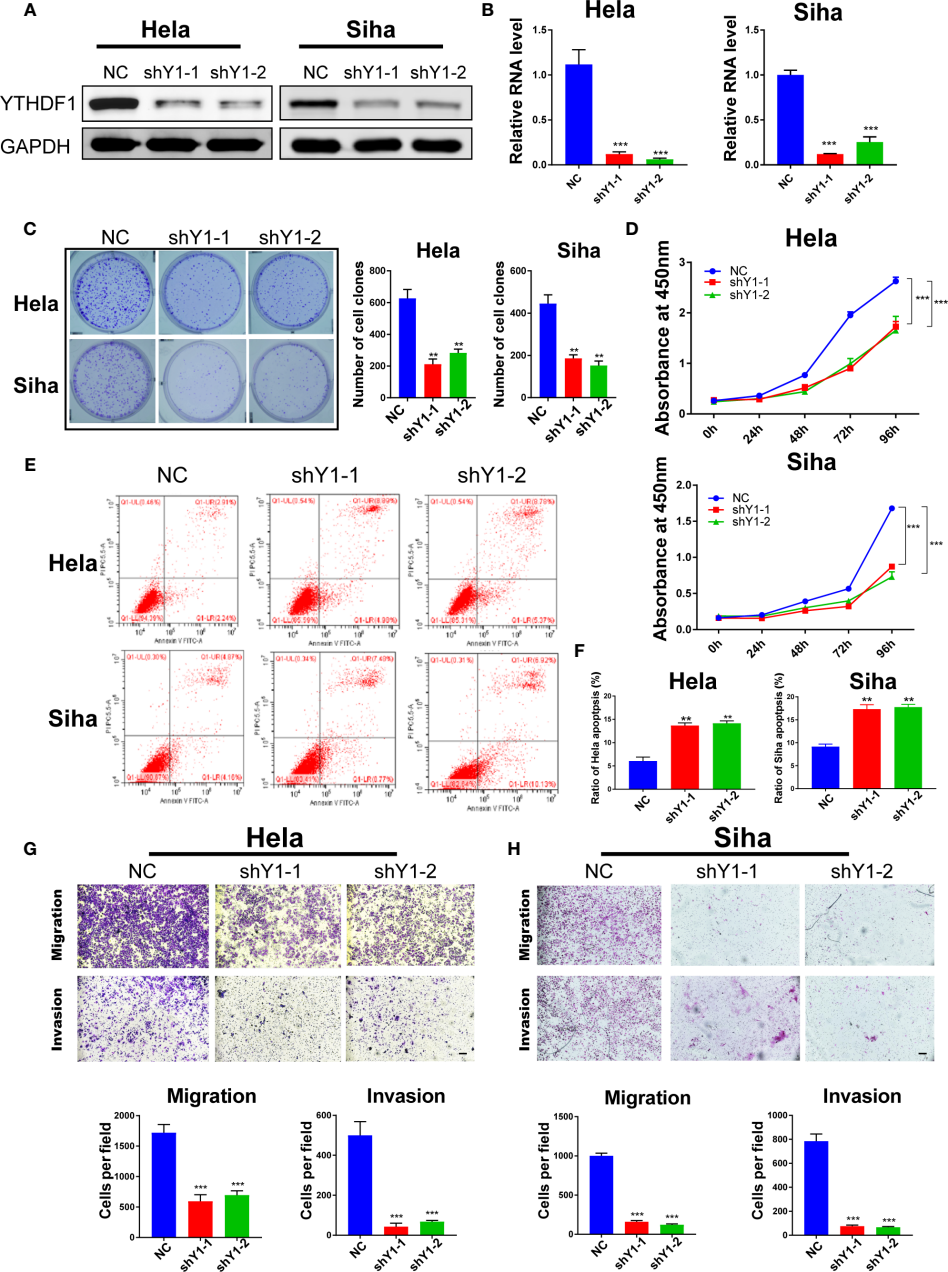
YTHDF1 regulates the proliferation, apoptosis, migration and invasion of cervical cancer cells *in vitro*. **(A, B)** YTHDF1 knockdown was confirmed in Hela and Siha cells by western blot and RT-qPCR. **(C)** Colony formation assays were performed using YTHDF1 knockdown cells and control. **(D)** Cell growth of cervical cancer cells upon YTHDF1 knockdown was detected by CCK-8 assay. **(E)** Apoptosis of cervical cancer cells upon YTHDF1 knockdown was detected by Annexin V/PI staining. **(F)** The quantitative analysis of apoptotic cells shown in **(E)**. **(G, H)** Transwell assays detecting migration and invasion of YTHDF1 knockdown cells as well as control cells. Scale bar, 200 μm. Data are shown as means ± S.D. **P < 0.01, ***P < 0.001.

To explore the function of YTHDF1 in cervical cancer, two shRNAs (shYTHDF1-1, shYTHDF1-2) were constructed and lentivirus was prepared to knock down YTHDF1. Western blot analysis and RT-qPCR results showed that YTHDF1 was effectively knocked down in both cervical cancer cells (**Figures 2A, B** **)**. Colony formation assays displayed that YTHDF1 knockdown decreased the colony formation ability of cervical cancer cells (**Figure 2C**). CCK-8 assays demonstrated that knocking down YTHDF1 substantially inhibited the proliferation of cervical cancer cells (**Figure 2D**). We also found that YTHDF1 knockdown induced apoptosis of cervical cancer cells (**Figures 2E, F** **)**. In addition, transwell assays revealed that the migration and invasion of cervical cancer cells were significantly suppressed upon YTHDF1 depletion (**Figures 2G, H** **)**. These results suggested that YTHDF1 promoted the proliferation, migration and invasion of cervical cancer cells, whereas inhibited apoptosis.

### YTHDF1 Deficiency Inhibits Tumorigenesis of Cervical Cancer Cells *In Vivo*

**Figure 3:**
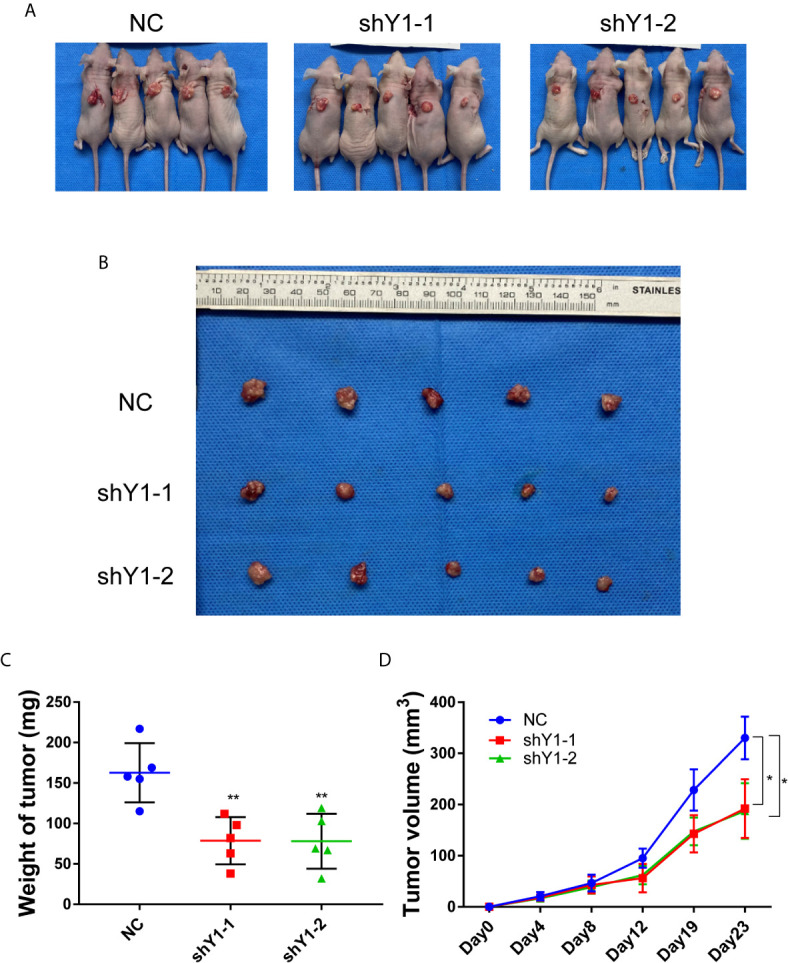
YTHDF1 deficiency inhibits tumorigenesis of cervical cancer cells *in vivo.* **(A, B)** Images of nude mice **(A)** and the isolated xenograft tumors **(B)** after sacrifice showed that the effect of YTHDF1 inhibition on the xenograft tumor growth of cervical cancer cells. **(C)** Tumor weight in each group was measured. **(D)** Tumor growth curves in nude mice were measured. Data are shown as means ± S.D. *P < 0.05, **P < 0.01.

In order to investigate the role of YTHDF1 in tumorigenesis *in vivo*, we conducted subcutaneous tumor formation experiments in nude mice. HeLa cells with YTHDF1 knockdown and control group were injected subcutaneously into nude mice, and tumor growth was monitored. Mice were sacrificed and tumors were isolated at 23 days post-injection (**Figure 3A**). As shown in the **Figure 3B**, the tumor volume of the YTHDF1 knockdown group was smaller than that of the control group. By recording the tumor growth curve and the weight at the time of sacrifice, we found that the average volume and weight of the tumors in the YTHDF1 knockdown group were markedly reduced compared with the control group (**Figures 3C, D** **)**. These results demonstrated that YTHDF1 had a cancer-promoting effect *in vivo*.

### Identification of Candidate Target Genes of YTHDF1 in Cervical Cancer

**Figure 4:**
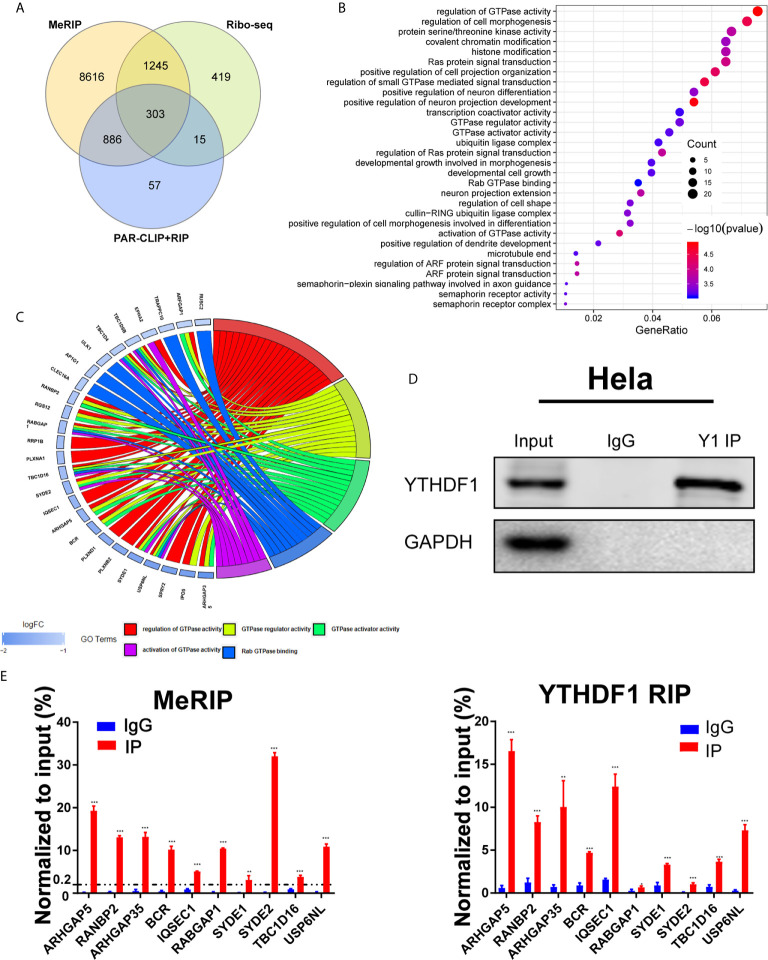
Identification of candidate targets of YTHDF1 in cervical cancer. **(A)** Overlapping analysis of genes from the results of meRIP-seq, PAR-CLIP/RIP-seq and Ribo-seq. **(B)** Gene ontology (GO) analysis of 303 genes. **(C)** Genes in GTPase activity regulation pathway. **(D)** RIP-PCR assays detecting the interactions between YTHDF1 and mRNAs of 10 candidate genes in Hela cells. IgG was used as an internal control. GAPDH was used as the negative control in western blot assays. **(E)** meRIP-PCR assays detecting the RNA m^6^A modification of 10 candidates in Hela cells. Data are shown as means ± S.D. **P < 0.01, ***P < 0.001.

To explore the underlying mechanisms of YTHDF1 in cervical cancer, we analyzed online meRIP-seq data (GSE46705), PAR-CLIP and RIP-seq data (GSE63591) to identify the targets of YTHDF1 in cervical cancer cells. According to Wang et al., YTHDF1 regulates gene expression by promoting RNA translation efficiency (8). Thus we also analyzed the ribosome sequencing (Ribo-seq) data in Hela cells upon YTHDF1 knockdown. Through overlapping the results of meRIP-seq, PAR-CLIP-seq, RIP-seq, and Ribo-seq, 303 genes were identified as the candidate targets of YTHDF1 (**Figure 4A**). After gene ontology (GO) analysis, these genes were related to GTPase activity regulation pathway significantly (**Figure 4B**). Thus, we ranked the genes involved in GTPase activity regulation pathway with down-regulated translation after YTHDF1 knockdown (**Figure 4C**) and selected the top 10 candidate genes for further study. We performed meRIP-PCR and RIP-PCR in Hela cells, and the results showed that all of these 10 candidate genes were subjected to m^6^A modification, and YTHDF1 could also bind to these transcripts (**Figures 4D, E** **)**.

### YTHDF1 Regulates RANBP2 Expression in Cervical Cancer

**Figure 5:**
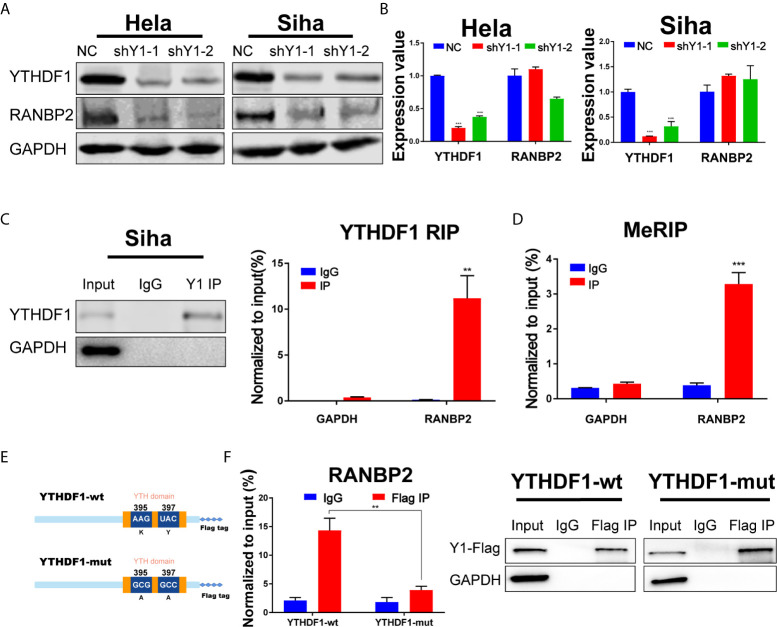
RANBP2 is the key target of YTHDF1 in cervical cancer. **(A)** Western blot detecting the protein level of RANBP2 in Hela and Siha cells upon YTHDF1 knockdown. **(B)** RT-qPCR detecting relative RNA level of RANBP2 in Hela and Siha upon YTHDF1 knockdown. **(C)** RIP-PCR assays detecting the interactions between YTHDF1 and RANBP2 mRNA in Siha cells. IgG was used as an internal control. GAPDH was used as the negative control in western blot assays. **(D)** meRIP-PCR assays detecting the m^6^A modification of RANBP2 mRNA in Siha cells. **(E)** Schematic of wild-type (YTHDF1-wt) and mutant (YTHDF1-mut) YTHDF1 constructs. **(F)** RIP-derived RNA and protein of wild-type (YTHDF1-wt) group and mutant (YTHDF1-mut) group in Hela cells were measured by RT-qPCR and western blot after immunoprecipitation by using the antibody specific to Flag, respectively. GAPDH was used as the negative control in western blot assays. Data are shown as means ± S.D. **P < 0.01, ***P < 0.001.

Among the 10 candidate targets of YTHDF1, RANBP2 was reported to be implicated in malignant progression of various cancers (23–25). Thus we detected the expression of RANBP2 upon YTHDF1 knockdown by western blot and found that YTHDF1 inhibition significantly reduced the protein expression of RANBP2 in both Hela and Siha cells (**Figure 5A**). RT-qPCR analysis showed that YTHDF1 knockdown did not affect the RNA level of RANBP2 (**Figure 5B**). Results of RIP-PCR and meRIP-PCR in Siha cells also revealed that YTHDF1 interacted with RANBP2 mRNA and m^6^A modification occurred on RANBP2 mRNA (**Figures 5C, D** **)**. To further investigate whether YTHDF1 regulated RANBP2 expression in an m^6^A-dependent manner, wide-type YTHDF1 (YTHDF1-wt) and m^6^A binding domain mutated YTHDF1 (YTHDF-mut) (**Figure 5E**) were ectopically expressed in Hela cells followed by RIP-PCR by using the antibody specific to FLAG. The results showed that mutation of m^6^A binding domain in YTHDF1 could substantially decrease the interaction between YTHDF1 and RANBP2 (**Figure 5F**). These results suggested that YTHDF1 regulates RANBP2 expression in an m^6^A-dependent manner.

### RANBP2 Plays an Oncogenic Role in Cervical Cancer Cells

**Figure 6:**
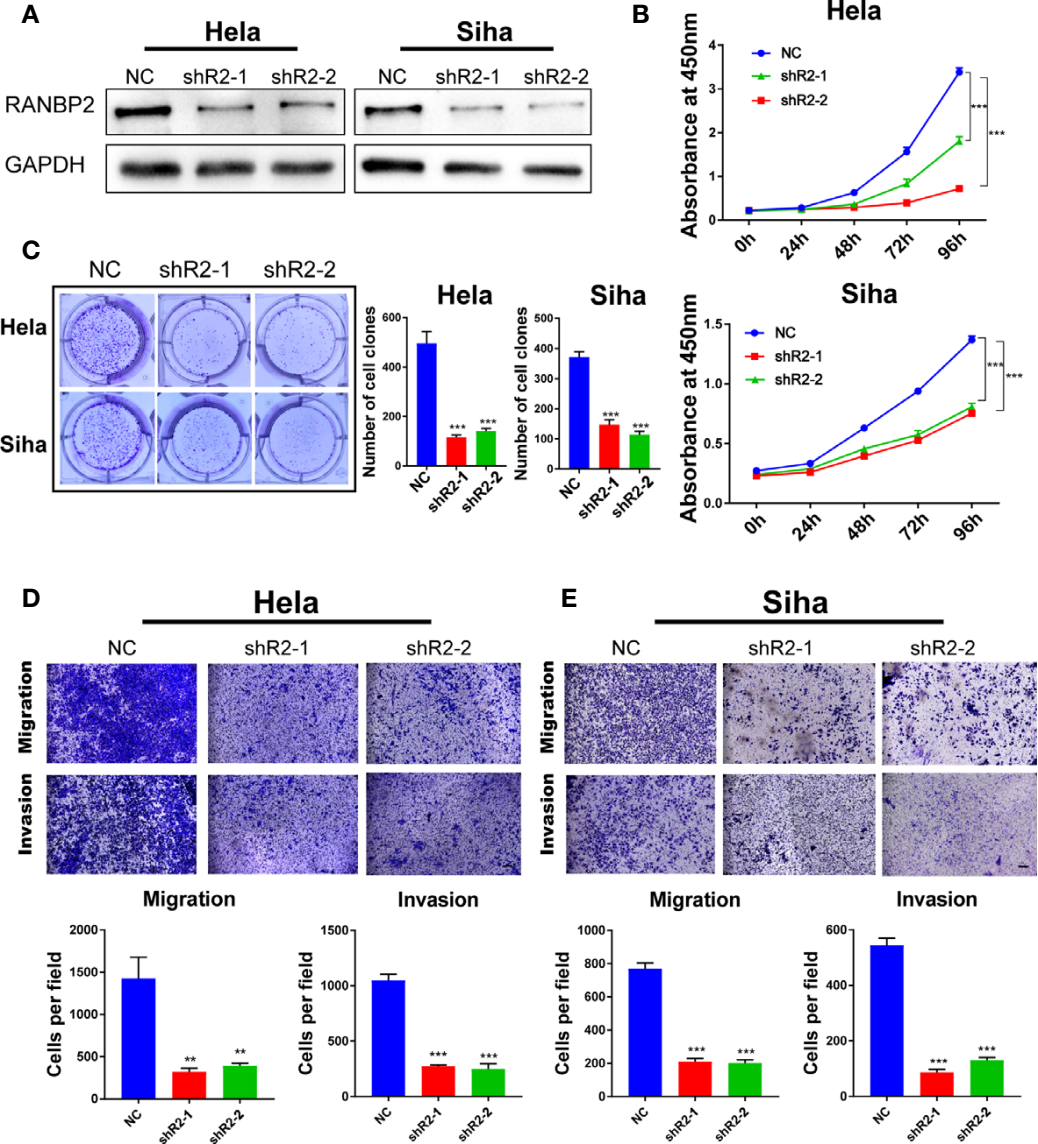
RANBP2 plays an oncogenic role in cervical cancer cells. **(A)** Detection of RANBP2 knockdown in Hela and Siha cell lines by western blot. **(B)** The effect of RANBP2 knockdown on cell growth was determined by CCK-8 assays. **(C)** Colony formation assays were performed in RANBP2 knockdown and control cells. **(D, E)** Migration and invasion assays of Hela and Siha cells upon RANBP2 knockdown. Scale bar, 200 μm. Data are shown as means ± S.D. **P < 0.01, ***P < 0.001.

**Figure 7:**
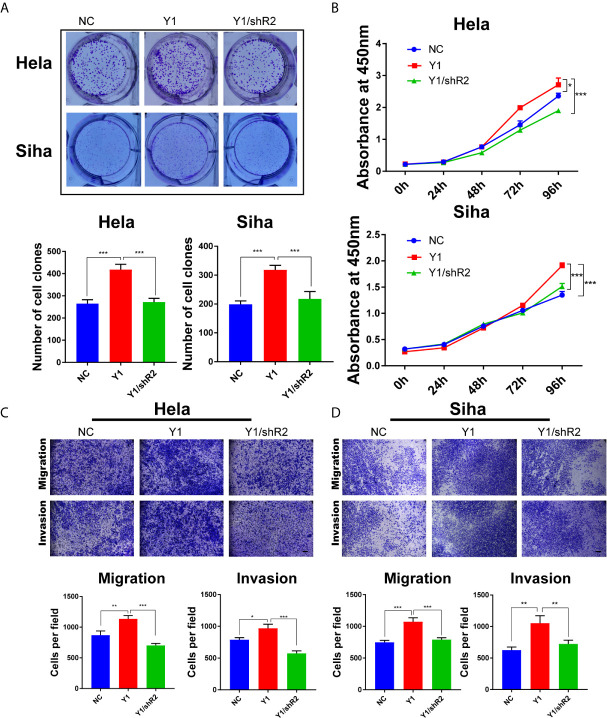
Knockdown of RANBP2 suppressed the proliferation, migration and invasion of YTHDF1-overexpressing Hela and Siha cells. **(A)** Colony formation assays were performed in YTHDF1-overexpressing Hela and Siha cells infected with the RANBP2 shRNA or controls. **(B)** The proliferation ability of YTHDF1-overexpressing Hela and Siha cells upon RANBP2 knockdown was assessed by CCK-8 assays. **(C, D)** Migration and invasion YTHDF1-overexpressing Hela **(C)** and Siha **(D)** cells upon RANBP2 knockdown was detected by transwell assays. Scale bar, 200 μm. *P < 0.05,**P < 0.01, ***P < 0.001.

**Figure 8:**
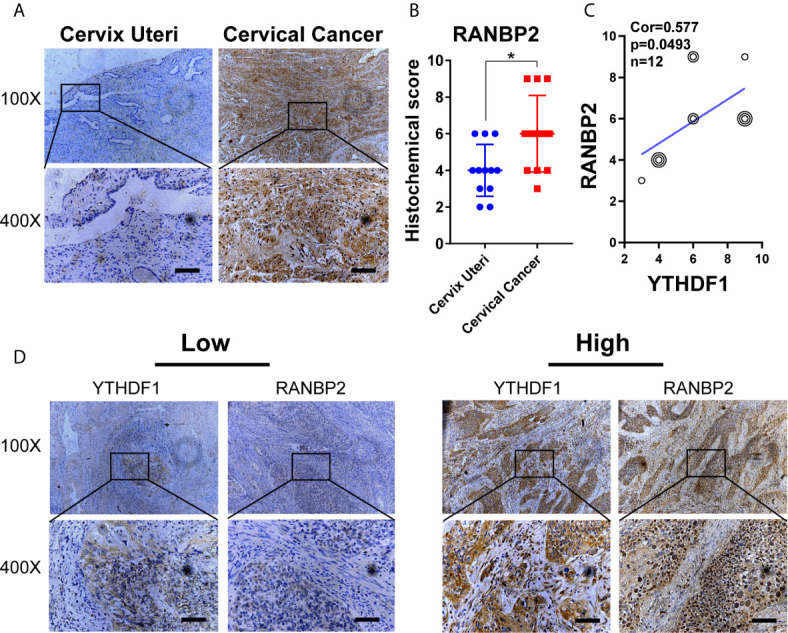
The expression of RANBP2 is positively correlated with YTHDF1 in cervical cancer. **(A)** Representative immunohistochemical images of RANBP2 protein expression in cervical cancer tissues and cervical epithelium tissues. Scale bar, 100 μm. **(B)** The quantitative analysis of RANBP2 expression in cervical cancer tissues and cervical epithelium tissues assessed by immunohistochemistry. **(C)** Spearman’s correlation analysis of RANBP2 and YTHDF1 expression in cervical cancer tissues. **(D)** Representative immunohistochemical images of YTHDF1 and RANBP2 in the cervical cancer tissues. Scale bar, 100 μm. Data are shown as means ± S.D. *P < 0.05.

To further examine the role of RANBP2 in cervical cancer cells, we designed two shRNAs targeting RANBP2. Western bot showed that RANBP2 was knocked down in both Hela and Siha cells (**Figure 6A**). CCK-8 assays showed that the proliferation of Hela and Siha cervical cancer cells was inhibited after RANBP2 knockdown (**Figure 6B**). Colony formation assays showed that knocking down RANBP2 markedly inhibited the colony formation ability of Hela and Siha cells (**Figure 6C**). Moreover, the results of transwell assays displayed that the migration and invasion abilities were significantly repressed upon RANBP2 knockdown in both Hela and Siha cells (**Figures 6D, E** **)**. Furthermore, the colony formation ability and proliferation of YTHDF1-overexpressing Hela and Siha cells were significantly increased, whereas knockdown of RANBP2 could compromise the effect of YTHDF1 overexpression on cervical cancer cells (**Figures 7A, B** **)**. Similarly, RANBP2 knockdown markedly suppressed migration and invasion of YTHDF1-overexpressing Hela and Siha cells (**Figures 7C, D** **)**. In addition, IHC results revealed that RANBP2 expression was higher in cervical cancer tissues compared to normal tissues (**Figures 8A, B** **)**. The correlation analysis suggested that there was a positive correlation between the protein expression of RANBP2 and YTHDF1 in cervical cancer (**Figures 8C, D** **)**. Collectively, these results suggested that YTHDF1-m^6^A-RANBP2 axis plays a significant role in cervical cancer.

## Discussion

Among female patients, cervical cancer ranks fourth in the world in terms of morbidity and mortality, and the fatality rate in developing countries is even higher. GLOBOCAN estimates that there were 570,000 women suffering from cervical cancer worldwide in 2018, and it caused about 311,000 deaths, of which about 85% of patients were from developing countries (1). Although immunotherapy has achieved a series of impressive results in the treatment of tumors, the effect of current treatment for advanced cervical cancer is not obvious (26). Therefore, it is urgent to explore the pathogenesis of cervical cancer and identify new targets for diagnosis and treatment. m^6^A modification is the most extensive internal modification of mRNA in eukaryotes and affects almost every aspect of RNA metabolism (27, 28). The reversibility of m^6^A is mainly achieved by the regulation of the methyltransferase (“writers”) and the demethylase (“erasers”). With the development of enzymology, the methyltransferases including METTL3, METL14, WTAP, etc (29, 30), and demethylases including FTO and ALKBH5, etc (31, 32) have been discovered. The m^6^A modification controls the fate of the modified RNA by interacting with different binding proteins (“readers”). The m^6^A binding protein is mainly a family of proteins containing the YTH domain, primarily including YTHDF1, YTHDF2, YTHDF3 (6, 8, 33) in the cytoplasm and YTHDC1 in the nucleus (34). It has been reported that m^6^A is closely related to various cancers (35). Compared with normal tissues, higher expression of METTL3 was found in human lung cancer and colon adenocarcinoma tissues (5, 14). METTL14 is highly expressed in acute myeloid leukemia cells and exerts its oncogenic role (36). FTO is highly expressed in acute myeloid leukemia, which could enhance the occurrence of leukemia, and inhibit the transretinoic acid-mediated differentiation of leukemia cells (37).

In cervical cancer, high-risk subtypes of the HPV are the cause in most cases (38). It has been reported that HPVs generated circRNAs encompassing the E7 oncogene (circE7), which was subjected to m^6^A modification, and promoted the proliferation of tumor cells (39). METTL3 promoted the stability of H2K through m^6^A modification, thereby promoting Warburg effect and the proliferation of cervical cancer cells (40). Here, we found that YTHDF1 is up-regulated in cervical cancer tissues and predicts the poor clinical outcomes. Through *in vitro* experiments we found that YTHDF1 depletion substantially inhibited the proliferation, migration and invasion of cervical cancer cells and promoted apoptosis. The subcutaneous tumor formation assays in nude mice also showed that YTHDF1 could promote the tumorigenesis of cervical cancer cells. These results suggest that YTHDF1 has an important tumor-promoting effect in cervical cancer. Therefore, m^6^A modification may play an important role in cervical cancer progression. Though hypomethylation of YTHDF1 promoter contributed to its high expression in cervical cancer, whether HPV infection regulates the expression of YTHDF1 or other m6A regulator requires more investigations and dissecting the relationship between HPV and m6A modification might be conducive to understanding the mechanisms underlying HPV-induced cervical cancer progression.

RANBP2 (RAN-binding protein 2), the largest nucleoporin in nuclear pore complexes (NPC) and the binding protein of RAN GTPase, is involved in mitosis and macromolecule transport (41). Ran GTPase regulates the ability of nuclear transport factors to bind and release cargo (42). The combination of RANBP2 and RanGTPase-activating protein (RanGAP1) promotes RAN GTPase-mediated nuclear export and nuclear import (43, 44). In Hela cells, absence of RANBP2 causes various mitotic abnormalities (45). In this study, YTHDF1 mediates the up-regulation of RANBP2 in an m^6^A-dependent manner in cervical cancer. The RANBP2-mediated RAN GTPase regulation has been implicated in the initiation and progression of several cancers. In liver cancer, SIRT1-mediated RANBP2 activation promotes progression of liver cancer (24). LIN28B cooperates with RANBP2 to promote RAN expression and activity of RAN GTPase, thereby driving the oncogenesis of neuroblastoma (25). In our study, RANBP2 promoted the growth, migration and invasion of cervical cancer cells. RANBP2 expression up-regulated by YTHDF1 might enhance the activity of RAN GTPase activity and aggravate the progress of cervical cancer, which might need more investigations in further study. Therefore, YTHDF1 might be a potential target for cervical cancer treatment.

## Conclusions

In conclusion, our study showed that the m^6^A “reader” YTHDF1 promotes the proliferation, migration and invasion of cervical cancer cells, and we also identified RANBP2 as the direct target of YTHDF1. YTHDF1 regulated RANBP2 translation in an m^6^A-dependent manner, which plays an important role in cervical cancer. YTHDF1 might represent a potential target for cervical cancer therapy.

## Data Availability Statement

The original contributions presented in the study are included in the article/**Supplementary Material**. Further inquiries can be directed to the corresponding authors.

## Ethics Statement

The studies involving human participants were reviewed and approved by Army military Medical University. The patients/participants provided their written informed consent to participate in this study. The animal study was reviewed and approved by Army military Medical University.

## Author Contributions

PY and TL put forward the ideas of this article. PY, TL and HW drafted and reviewed the article. HW, JK, QL, DY and XL performed the experiments. QW and YY helped with acquisition of data and analysis and interpretation of data. All authors contributed to the article and approved the submitted version.

## Funding

This work was sponsored by the Natural Science Foundation of Chongqing, China (cstc2020jcyj-msxmX0344), the National Natural Science Foundation of China (81902668) and the National Key R&D Program of China (2018YFC1313400).

## Conflict of Interest

The authors declare that the research was conducted in the absence of any commercial or financial relationships that could be construed as a potential conflict of interest.
