# Supplementary material for: YTHDF1 Aggravates the Progression of Cervical Cancer Through m6A-Mediated Up-Regulation of RANBP2
Source: Front Oncol. 2021 Mar 19;11:650383. doi: 10.3389/fonc.2021.650383 (PMC8017305; doi:10.3389/fonc.2021.650383)
Supplement: Supplementary file 1 [file DataSheet_1.docx]

**Supplement Table 1.** Clinicopathological characteristics of cervical cancer patients.

| **Characteristic** | **Number of cases** |
| --- | --- |
| **Age (years)** |  |
| ≤ 44 | 2 |
| > 44 | 10 |
| **FIGO stage**  I  II  III  IV | 0  2  9  1 |
| **Tumor size, cm** |  |
| < 4 | 9 |
| ≥ 4 | 3 |
|  |  |
| **Pelvic lymph node metastasis** |  |
| No | 9 |
| Yes | 3 |
|  |  |
| **Vital status (at last follow-up)** |  |
| Alive | 7 |
| Dead | 5 |
| **Differentiation grade** |  |
| G1 | 1 |
| G2 | 6 |
| G3 | 5 |
| **Deep Myometrium invasion** |  |
| No | 0 |
| Yes | 12 |
|  |  |
| **Expression of YTHDF1 protein** |  |
| Low | 6 |
| High  **Chemotherapy**  No  Yes | 6  5  7 |

**Supplement Table 2.** Primers and shRNA seqence

|  | Name | Oligo sequence |
| --- | --- | --- |
| shRNA target oligo | shYTHDF1-1 | GATACAGTTCATGACAATGA |
|  | shYTHDF1-2 | GAAACGTCCAGCCTAATTCT |
|  | shRANBP2-1 | CGGCGAAGTGATGATATGTTT |
|  | shRANBP2-2 | CCTTGCATGGTGTGAAATAAA |
| Primers used in PCR | YTHDF1-F | ATGTCGGCCACCAGCGTGGACA |
|  | YTHDF1-R | TCATTGTTTGTTTCGACTCTGC |
|  | GAPDH-F | TCTTAAGAAGACGACGGCTTCAG |
|  | GAPDH-R | TTGCTCTCTCACTTGTCCTCGAT |
|  | RANBP2-F | AAACCTCCGATTGCAGCTCAT |
|  | RANBP2-R | GGCAAAGATGGCCTTAATCCT |
|  | ARHGAP5-F | AGGGAAGCTCAACGTAGATGG |
|  | ARHGAP5-R | ATGATCCACGCATTCATCACAT |
|  | SYDE2-F | GGTCCTCTGTGATACGCAGTG |
|  | SYDE2-R | CGGGCACGACCCTTCATTC |
|  | SYDE1-F | GGGAGCCTACCTGCAAAGC |
|  | SYDE1-R | GCCCTAAAGAGGTGTCCTCAG |
|  | BCR-F | TACCAGAGCATCTACGTCGGG |
|  | BCR-R | CCTCCGCAATCCTCAAAACTC |
|  | TBC1D16-F | TCCAAGAACAATGTCTGCGTG |
|  | TBC1D16-R | GCTCCCAGCATCTCATCCTT |
|  | ARHGAP35-F | AGTGTGGTGGGATTATCTGGG |
|  | ARHGAP35-R | CGAAGCGGTTGCACAAACAA |
|  | IQSEC1-F | AGAGAACCGCATGTCACGC |
|  | IQSEC1-R | CTTCCCCTCGAAGTAGGAGC |
|  | USP6NL-F | GTGGAACGGCAAAAGCACC |
|  | USP6NL-R | AGCTGGAGTGGTATTCCTTTGTA |
|  | RABGAP1-F | ACAGGACTCAAGATTGTAGGGAA |
|  | RABGAP1-R | CTGGTCGTCCATTGGAGGA |
|  |  |  |
